# Insight and self-esteem in patients with bipolar disorders

**DOI:** 10.1192/j.eurpsy.2021.511

**Published:** 2021-08-13

**Authors:** S. Hentati, M. Ben Abdallah, M. Daoud, I. Baati, R. Sallemi, I. Feki, J. Masmoudi

**Affiliations:** 1 Psychiatry A, hedi chaker hospital, Sfax, Tunisia; 2 Department Of Psychiatry (a), CHU Hedi CHaker hospital Sfax Tunisia, Sfax, Tunisia; 3 Psychiatry A, CHU Hédi Chaker Sfax, Sfax, Tunisia; 4 Psychiatry, Hedi chaker hospital, Sfax, Tunisia

**Keywords:** insight, self-esteem, Bipolar Disorders

## Abstract

**Introduction:**

The recurrence of bipolar disorder due to poor treatment adherence can be explained by different factors.The poor awareness of the disorder seems to be the major cause.

**Objectives:**

To evaluate insight in patients followed for euthymic bipolar disorders and determine the factors correlated with it,mainly the self-esteem.

**Methods:**

A cross-sectional descriptive and analytical study of 33 euthymic subjects with bipolar I and II disorders (DSM 5) and followed up at the psychiatric consultation in Hédi Chaker university Hospital of Sfax.Data collection was performed using a sheet exploring socio-demographic and clinical data. We used Birchwood insight scale to assess the quality of insight and Rosenberg’s Self-Esteem scale.

**Results:**

The average age of our patients was 44,52±12,99 years old.The sex ratio =0.32.Patients were followed for bipolar I disorder(60.6%).The first episode of the disease was depressive in(51.5%) of cases.The average number of depressive episodes was1.97±1.87.The last episode was depressive in(57.6%) or manic in(42.4%).There were no psychotic characteristics in(42.4%) of cases.The patients had good insight in(54.5%).The average of self-esteem score was27±7.85and it was low in 51.5% of cases.Factors correlated with good insight were bipolar II disorder(p=0.001), high number of depressive episodes(p=0.013) and absence of psychotic characteristics(p=0.003) during the last episode.In addition,good insight was significantly associated with low self-esteem(p=0.023).

**Conclusions:**

Our study shows that a poor insight depends mainly on the clinical characteristics of bipolar disorders. Moreover,low self-esteem seems to be linked to it. For this reason, our attention should be focused on psychoeducation to improve insight, especially during episodes, in order to facilitate integration and increase patients’ self-esteem.

